# Iatrogenic Ulnar Nerve Injury Post–K‐Wire Fixation of Supracondylar Humeral Fracture in a Child With Ulnar Nerve Instability: A Case Report and Review of the Literature

**DOI:** 10.1155/cro/4487507

**Published:** 2026-03-08

**Authors:** Mohamed F. Mostafa, Kishan N. Paralaya, Mohamed I. El-Husseini

**Affiliations:** ^1^ Department of Orthopaedic Surgery, Kalba Hospital, Emirates Health Services, Sharjah, UAE

**Keywords:** iatrogenic, instability, supracondylar humeral fracture, ulnar nerve

## Abstract

**Background:**

Iatrogenic ulnar nerve damage is not an uncommon consequence after K‐wire fixation of supracondylar humeral fractures in children. The risk of injury may be increased when the ulnar nerve is hypermobile.

**Case Report:**

We present a 7‐year‐old boy who underwent closed reduction and crossed K‐wire fixation for a Gartland Type IIb supracondylar fracture of the right humerus. The ulnar nerve was not palpable in the cubital tunnel but felt as a cord‐like structure in front of the medial epicondyle. Immediately following surgery, the patient developed paresthesia and painful incomplete clawing of the ring and little fingers. Exploration on the second day of surgery revealed the ulnar nerve anterior to the medial epicondyle and tented over the K‐wire. The K‐wire was replaced with one more lateral pin, and the nerve was decompressed. Complete recovery of nerve injury was noticed 4weeks after exploration.

**Conclusion:**

Given the greater prevalence of ulnar nerve instability among children, it is prudent to preoperatively evaluate patients for evidence of ligament laxity or ulnar nerve instability of the contralateral normal elbow. Prompt exploration and K‐wire removal can confirm nerve continuity, reassure the family, and ensure swift recovery from nerve injury.

## 1. Introduction

Supracondylar humeral fractures represent more than 60% of all elbow fractures in children aged 5–7 years [1–3]. According to the Wilkins‐modified Gartland classification, Type IIb and III fractures are considered unstable and should be managed surgically by closed reduction and percutaneous pinning [4, 5]. A crossed Kirschner‐wire (K‐wire) configuration using lateral and medial pins remains the most biomechanically stable fixation method [6, 7]. However, inserting the medial pin carries a significant risk of iatrogenic injury to the ulnar nerve [8, 9]. This concern has led to the innovation of a lateral‐only configuration that utilizes three lateral pins as proposed by Zionts et al. [6], two diverging lateral pins described by Skaggs et al. [10], and two converging lateral pins in Dorgan′s method [11]. Although most displaced supracondylar humeral fractures can be sufficiently stabilized with lateral pins, certain fracture patterns such as Type III fractures with medial cortex comminution and initial cubitus varus deformity still necessitate a medial pin for optimal stability [12, 13].

Ulnar nerve instability refers to the recurrent subluxation or dislocation and relocation of the ulnar nerve when the elbow is flexed and extended, respectively. The tip of the medial epicondyle is used to determine the location of the ulnar nerve. The nerve moves and stops at the tip of the medial epicondyle in subluxation, while it slides anteriorly in dislocation [14]. The condition is more common than generally thought with reported incidence of 10% to–31%, and it is bilateral in more than half of cases [15–17]. Instability of the ulnar nerve at the elbow may increase the chance of iatrogenic damage during medial pinning. Knowing the precise location of the ulnar nerve in relation to the medial epicondyle can assist the surgeon in avoiding this complication. We report a case of iatrogenic ulnar nerve injury after closed reduction and K‐wire fixation in a child with ulnar nerve dislocation.

## 2. Case Report

A 7‐year‐old otherwise healthy boy experienced a witnessed fall on his outstretched hand while playing soccer and arrived at the emergency room with pain and swelling of his right elbow. Radiographs confirmed a closed supracondylar fracture right humerus, Gartland Type I. The patient was managed conservatively with a long arm splint. After the splint was taken off at home a few days later, the patient sustained another fall injury but more severe than the previous one. He developed a progression of the fracture to Gartland Type IIb. He was then admitted for closed reduction and crossed 2 mm K‐wire fixation. After verifying the accurate reduction with the image intensifier by rotating the C‐arm from anteroposterior to lateral view, the lateral pin was inserted first. During medial pinning, the elbow was stretched to less than 90° of flexion. The ulnar nerve was impalpable in the cubital tunnel. Instead, a cord‐like structure was palpable just anterior to the medial epicondyle, which was protected with the thumb of the free hand while inserting the medial pin. Fracture reduction and position of K‐wires were checked with an image intensifier. The limb was immobilized in a long back slab with the elbow flexed less than 90° and the forearm in a neutral position.

### 2.1. Clinical Findings

**Figure 1:**
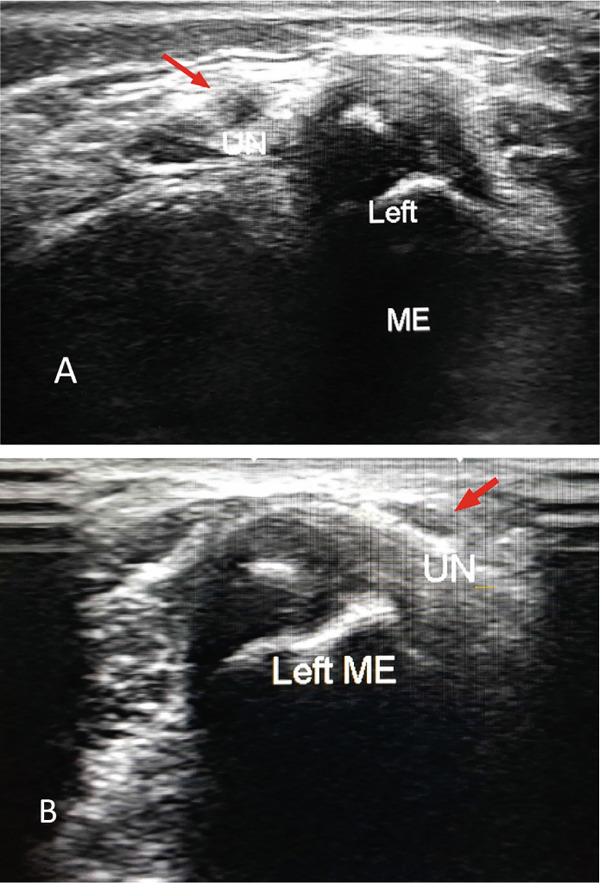
Axial ultrasound image of the contralateral normal elbow; (A) the ulnar nerve (red arrow) lying just posterior to the tip of the medial epicondyle on elbow extension, (B) the ulnar nerve (red arrow) dislocated anterior to the medial epicondyle on elbow flexion.

Immediately postoperatively, the patient experienced increasing paresthesia, altered sensation in the ulnar nerve distribution, and painful incomplete clawing of the little and ring fingers. These symptoms and signs of ulnar nerve injury were not present before surgery and did not recover over the hours following surgery. The ulnar nerve location in the contralateral elbow was evaluated clinically. It was not detectable in the cubital tunnel and shifted over the medial epicondyle to a more anterior position when the elbow was flexed. Real‐time ultrasound examination of the normal elbow using Toshiba Aplio 400 with a 10 MHz high‐frequency linear array transducer confirmed ulnar nerve instability. The probe was applied on an imaginary line connecting the medial epicondyle and olecranon process and stabilized against the medial epicondyle during elbow flexion (Figure 1). The patient did not show any of the five Wynne‐Davies signs of ligamentous laxity [15]. The family was understandably concerned about the possible ulnar nerve injury of their child.

### 2.2. Therapeutic Intervention

**Figure 2:**
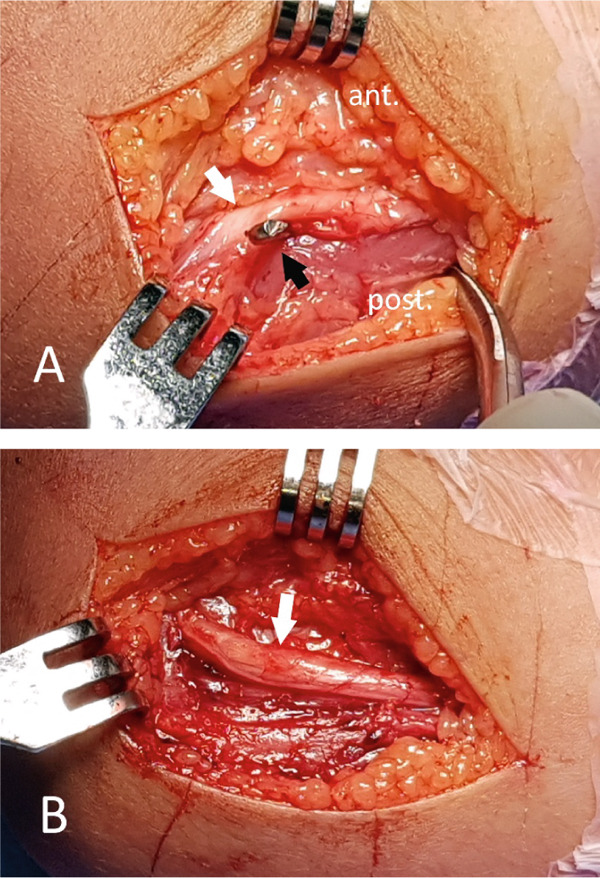
Intraoperative photo; (A) the ulnar nerve (white arrow) located anterior to the medial epicondyle, tented over the K‐wire (black arrow) and looked devascularized, (B) the nerve (white arrow) regained vascularity and thickness after removal of the K‐wire and soft tissue decompression.

On the second postoperative day, the patient underwent exploration of the ulnar nerve. The nerve was located in front of the medial epicondyle and tented over the K‐wire (Figure 2a). It was otherwise in continuity and not penetrated nor tethered by the K‐wire. Extension of the elbow caused more stretch of the nerve over the wire to appear blanched and devascularized. An additional lateral pin was inserted to maintain reduction and augment fixation. The medial pin was removed gently while protecting the nerve. All fibrous tissue coverings of the nerve in the cubital tunnel and between the two heads of flexor carpi ulnaris were released. After removal of the medial pin and soft tissue decompression, the nerve immediately regained vascularity (Figure 2b). The nerve was then left in its relaxed position and the wound was closed in layers. The limb was splinted in a long arm back slab.

### 2.3. Follow‐Up Outcomes

The patient noticed complete relief of pain and numbness in the ring and little fingers on the night of surgery and recovery of all types of sensation, including proprioception and discriminative touch, by the second day. Four weeks after surgical exploration, the patient reported complete restoration of ulnar nerve motor and sensory function. The lateral K‐wires were taken out after 6 weeks. At the final follow‐up, the patient showed a full range of motion of the fingers, forearm, and elbow.

## 3. Discussion

The most popular procedures for managing displaced supracondylar humeral fractures in pediatric patients are closed reduction and percutaneous pinning. Although a crossed K‐wire configuration provides the highest fracture stability by resisting axial rotation, the medial pin poses a potential risk of iatrogenic injury to the ulnar nerve, ranging from 2% to 20% [18, 19]. This has led some surgeons to develop and prefer the lateral‐only configuration. Research has demonstrated that lateral‐only pinning yields comparable outcomes to crossed K‐wire fixation in terms of instability and the rate of nerve injury [10, 20]. Nevertheless, the authors maintain that crossed pinning reduces the chance of losing the reduction, particularly in unstable Type III and Type IV fractures. It is crucial to minimize iatrogenic ulnar nerve injury during insertion of the medial pin by extending the elbow, palpating and safeguarding the ulnar nerve within the cubital tunnel, or creating a small incision at the medial epicondyle [13, 21–23].

Ulnar nerve instability in the elbow is relatively common in children with a reported incidence of up to 31% [15–17]. The likely reason for this condition is a congenital laxity of the supporting ligaments. According to Zaltz et al. [15], among children exhibiting all five Wynne‐Davis signs of ligamentous laxity, 25% have dislocation and 76% have subluxation of the ulnar nerve. However, ulnar nerve instability at the elbow can occur in the absence of ligamentous laxity and be caused by dysplasia of the retrocondylar groove, prominent medial head of triceps, and the presence of anconeus epitrochlearis pushing the ulnar nerve from its sulcus on flexing the elbow [24, 25]. We could not find any evidence of ligament laxity in our case. Bilateral ulnar nerve instability has been reported in more than half of patients [15–17]. Dynamic ultrasound examination of the contralateral uninjured elbow revealed instability of the ulnar nerve to be bilateral in the current case.

The clinician must recognize that in children approximately one third of the ulnar nerve may be situated at the tip or in front of the medial epicondyle on elbow flexion. This could increase the likelihood of iatrogenic injury to the ulnar nerve during medial pinning. Wilkins et al. [26] reported that inserting the medial pin through the tip of the medial epicondyle carries a chance of damaging the ulnar nerve if the nerve is hypermobile. Rasool [8] noted in one out of six instances of iatrogenic ulnar nerve injury due to medial pin placement, the nerve was hypermobile and found fixed anterior to the medial epicondyle. Knowing the location of the ulnar nerve is a paramount step before medial pinning. Soldado et al. [27] used ultrasound to guide medial pinning and concluded that although it is a challenging technique, ultrasound assistance might be beneficial for avoiding ulnar nerve injury. Assessing both pin placement and the ulnar nerve location using coronal‐sectional ultrasound with the elbow flexed at 90° is technically challenging. Wu et al. [28] used a modified oblique ultrasound plane with the elbow extended to provide real‐time monitoring during pin insertion, enhance visualization, and prevent ulnar nerve subluxation. However, ultrasound is operator‐dependent, and proficiency in musculoskeletal ultrasound is crucial for the effective utilization of this technique. The position of the elbow during K‐wire fixation of supracondylar humeral fracture is also important. Swenson [29] initially proposed pinning on a sharply flexed elbow to achieve and maintain reduction of the distal fragment. However, this is no longer advised because of the high incidence of anterior subluxations of the ulnar nerve over the medial epicondyle when the elbow is flexed beyond 90°. The elbow must therefore be in relative extension when performing medial pinning. The surgeon can easily adjust the remaining extension of the distal fragment and maintain reduction simply by applying an anteriorly directed force with the thumb of the free hand while inserting the K‐wire. Despite performing medial pinning with the elbow in less than 90° flexion, we could not locate the ulnar nerve in the cubital tunnel instead the nerve was felt as a cord‐like structure at the tip or just in front of the medial epicondyle. It is thought that iatrogenic injury to the ulnar nerve almost always occurs due to an improperly placed pin with the starting point inferior and posterior to the medial epicondyle. Even with ideal insertion of the medial pin through the apex of the medial epicondyle, the ulnar nerve is still liable to injury if it is hypermobile [8].

Almost all of the literature uses a clinical approach to diagnose iatrogenic ulnar nerve injury. The authors described the injury as purely sensory, a combination of motor and sensory, or as complete or partial [8, 18, 21]. Progressive paresthesia and painful partial clawing on extension of the ring and little fingers were the most evident clinical findings early postoperative in the present case. Currently, there is no definite imaging technique available to determine whether iatrogenic nerve injury has occurred. Ultrasound has been utilized to determine if the nerve maintains continuity [30]. However, the effectiveness of ultrasound varies, as the nerve may still appear continuous while either being damaged or tethered, conditions that might not be accurately revealed. Nerve conduction studies can be conducted to verify the existence of a nerve injury, but will not indicate the injury′s cause, whether it was iatrogenic or associated with nerve entrapment at the fracture site [18].

The treatment options for ulnar nerve damage following percutaneous pinning are hotly debated. The lack of information in the literature about how to treat this complication to help clinicians make decisions presents a clinical and medicolegal dilemma. Some authors advocated early extraction of medial K‐wire, surgical exploration of the nerve, and K‐wire repositioning [8, 31, 32], whereas others prefer a more conservative strategy, suggesting that the K‐wire should be removed once union has been achieved, assuming that full recovery is likely regardless of the underlying cause [9, 33, 34]. Although the majority of authors expectantly treated iatrogenic ulnar nerve palsies, this was frequently because the complication was not discovered until the wires were taken out after 3–6 weeks. Because neuropraxia or axonotmesis are the most common types of injury, it is generally agreed that the injury resolves within 6 months [2]. However, there are many cases reported without full recovery at final follow‐up [8, 18, 21]. The incomplete recovery aligns with the findings of Mohler and Hanel, which indicate that although the ulnar nerve has moderate regenerative capabilities, the level of injury considerably influences the outcome [35]. Other research has proposed that aside from injury level, delays in surgical exploration can lead to prolonged periods before returning to normal function [31]. Ulnar nerve injury after medial pinning was found on exploration to be due to direct penetration by K‐wire, tethering from high‐spun wire, cubital tunnel constriction, or a hypermobile nerve positioned over the medial epicondyle [8, 9, 18, 31]. In the current case, surgical exploration was performed within 12 h of injury. The nerve was discovered anterior to the medial epicondyle and tented over the wire. Full recovery was achieved 4 weeks after exploration and K‐wire removal.

## 4. Conclusion

Iatrogenic injury to the ulnar nerve is a recognized risk of percutaneous K‐wire fixation of supracondylar humeral fractures in pediatric patients. The risk is increased in the presence of ulnar nerve instability. This complication should not diminish the effectiveness of the crossed‐pinning technique in obtaining a stable fixation construct and all measures should be taken to avoid this complication. In the setting of increased frequency of ulnar nerve instability among children, the authors would advise preoperative assessment of the patients for evidence of ligament laxity or ulnar nerve instability of the contralateral uninjured elbow. If ulnar nerve instability is highly suspected preoperatively and confirmed intraoperatively by the impalpable nerve in the cubital tunnel, the choice will be either to proceed with lateral‐only pinning or to make a small incision medially with exploration of the ulnar nerve based on the fracture stability. Although most of the literature supports that ulnar nerve injuries generally heal over time, this is not always guaranteed. The authors advocate for the prompt extraction of the K‐wire, exploration, nerve decompression, and refixation if required. This approach ensures nerve continuity, provides an explanation for the symptoms, reassures the family, and promotes a quicker recovery from the nerve injury.

## Funding

No funding was received for this manuscript.

## Consent

Written informed consent was obtained from the patient and guardians for personal data processing and publication of this report.

## Conflicts of Interest

The authors declare no conflicts of interest.

## Data Availability

The data that support the findings of this study are available from the corresponding author upon reasonable request.
